# Serum Neurofilament Light Chain in Replication Factor Complex Subunit 1 CANVAS and Disease Spectrum

**DOI:** 10.1002/mds.29680

**Published:** 2023-12-06

**Authors:** Ilaria Quartesan, Elisa Vegezzi, Riccardo Currò, Amanda Heslegrave, Chiara Pisciotta, Pablo Iruzubieta, Alessandro Salvalaggio, Gorka Fernández‐Eulate, Natalia Dominik, Bianca Rugginini, Arianna Manini, Elena Abati, Stefano Facchini, Katarina Manso, Ines Albajar, Rhiannon Laban, Alexander M. Rossor, Anna Pichiecchio, Giuseppe Cosentino, Paola Saveri, Ettore Salsano, Francesca Andreetta, Enza M. Valente, Henrik Zetterberg, Paola Giunti, Tanya Stojkovic, Chiara Briani, Adolfo López de Munain, Davide Pareyson, Mary M. Reilly, Henry Houlden, Cristina Tassorelli, Andrea Cortese

**Affiliations:** ^1^ Department of Brain and Behavioral Sciences University of Pavia Pavia Italy; ^2^ IRCCS Mondino Foundation Pavia Italy; ^3^ Department of Neuromuscular Diseases UCL Queen Square Institute of Neurology London United Kingdom; ^4^ Department of Neurodegenerative Disease UCL Queen Square Institute of Neurology London United Kingdom; ^5^ UK Dementia Research Institute at UCL London United Kingdom; ^6^ Fondazione IRCCS Istituto Neurologico Carlo Besta Milan Italy; ^7^ Neurology Department, Donostia University Hospital Osakidetza, and Biodonostia Health Research Institute‐UPV‐EHU San Sebastián Spain; ^8^ Department of Neuroscience University of Padova Padova Italy; ^9^ Neuro‐myology Department, Institut de Myologie, Pitié‐Salpêtriére Hospital APHP, Sorbonne University Paris France; ^10^ Department of Neurology and Laboratory of Neuroscience IRCCS Istituto Auxologico Italiano Milan Italy; ^11^ Department of Pathophysiology and Transplantation (DEPT) University of Milan Milan Italy; ^12^ Clinical Neurochemistry Laboratory Sahlgrenska University Hospital Mölndal Sweden; ^13^ Department of Psychiatry and Neurochemistry, Institute of Neuroscience and Physiology The Sahlgrenska Academy at the University of Gothenburg Mölndal Sweden; ^14^ Hong Kong Center for Neurodegenerative Diseases Clear Water Bay Hong Kong China; ^15^ Wisconsin Alzheimer's Disease Research Center, University of Wisconsin School of Medicine and Public Health, University of Wisconsin‐Madison Madison Wisconsin USA; ^16^ Department of Clinical and Movement Neuroscience UCL Queen Square Institute of Neurology London United Kingdom

**Keywords:** ataxia, biomarkers, cerebellar ataxia neuropathy vestibular areflexia syndrome, neurofilament light chain, replication factor complex subunit 1

## Abstract

**Background:**

Biallelic intronic AAGGG repeat expansions in the replication factor complex subunit 1 (*RFC1*) gene were identified as the leading cause of cerebellar ataxia, neuropathy, vestibular areflexia syndrome. Patients exhibit significant clinical heterogeneity and variable disease course, but no potential biomarker has been identified to date.

**Objectives:**

In this multicenter cross‐sectional study, we aimed to evaluate neurofilament light (NfL) chain serum levels in a cohort of *RFC1* disease patients and to correlate NfL serum concentrations with clinical phenotype and disease severity.

**Methods:**

Sixty‐one patients with genetically confirmed *RFC1* disease and 48 healthy controls (HCs) were enrolled from six neurological centers. Serum NfL concentration was measured using the single molecule array assay technique.

**Results:**

Serum NfL concentration was significantly higher in patients with *RFC1* disease compared to age‐ and‐sex‐matched HCs (*P* < 0.0001). NfL level showed a moderate correlation with age in both HCs (*r* = 0.4353, *P* = 0.0020) and patients (*r* = 0.4092, *P* = 0.0011). Mean NfL concentration appeared to be significantly higher in patients with cerebellar involvement compared to patients without cerebellar dysfunction (27.88 vs. 21.84 pg/mL, *P* = 0.0081). The association between cerebellar involvement and NfL remained significant after controlling for age and sex (β = 0.260, *P* = 0.034).

**Conclusions:**

Serum NfL levels are significantly higher in patients with *RFC1* disease compared to HCs and correlate with cerebellar involvement. Longitudinal studies are warranted to assess its change over time.

Biallelic AAGGG repeat expansions in intron 2 of the gene encoding replication factor complex subunit 1 (*RFC1*) were identified as the cause of cerebellar ataxia with neuropathy and vestibular areflexia syndrome (CANVAS) and disease spectrum (here shortened as *RFC1* disease).^1^

Affected individuals exhibit significant clinical heterogeneity, starting with an isolated sensory neuropathy, with or without chronic cough, and progressing to a more complex ataxia with cerebellar dysfunction in later disease stages.^2^, ^3^ Bilateral vestibular areflexia is also often present but can be easily overlooked if not tested for. The clinical manifestations of *RFC1* disease have expanded beyond the classical CANVAS to encompass dysautonomia, parkinsonism, and cognitive impairment.^4^, ^5^, ^6^

Multiple studies have established that neurofilament light (NfL), a component of the axonal cytoskeleton released into the cerebrospinal fluid (CSF) and blood stream after neuronal damage,^7^ is a promising fluid biomarker for neurodegenerative conditions, including Charcot–Marie–Tooth disease, hereditary transthyretin (ATTR) amyloidosis, and hereditary ataxias.^8^, ^9^, ^10^, ^11^, ^12^, ^13^ Increased CSF or serum NfL levels in patients carrying biallelic *RFC1* expansions have been observed in two single cases.^14^, ^15^ However, there is still scant evidence of its diagnostic role or its ability to reflect progression of the condition over time.

In this multicenter cross‐sectional study, we aimed to assess whether serum NfL may represent a potential biomarker for *RFC1* disease using ultrasensitive single molecule array (Simoa) immunoassay technology. Furthermore, we sought to evaluate the correlation between NfL concentration and clinical phenotype and severity.

## Patients and Methods

Serum samples (n = 50) were prospectively collected, after informed consent, from patients with a genetically confirmed diagnosis of *RFC1* disease^1^ and consecutively attending neurology clinics in six neurological centers (London: 10; Pavia: 16; Milan: 1; Paris: 7; Padova: 7; San Sebastian: 9). In 11 patients, serum was retrieved from the biorepository (Milan).

Patients' neurological history and clinical signs, including the presence of sensory neuropathy, cerebellar dysfunction (defined by the presence of one or more of the following signs: broken pursuits, dysmetric saccades, gaze‐evoked or downbeat nystagmus, dysarthria, and dysphagia), vestibular areflexia, dysautonomia, cognitive impairment, and parkinsonism, were collected based on a standard template. Patients with other neurologic diseases were excluded. In prospectively collected data, neurological examinations were performed at serum collection. In the 11 patients whose serum was retrieved from biorepositories, examination was performed at a mean of 15 months from serum sampling (ranging from 24 months before sampling to 12 months after sampling).

Based on the known progression of the disease from an isolated sensory neuropathy to a complex neuropathy with cerebellar dysfunction or full CANVAS,^3^ patients were divided into two clinical subtypes as a proxy of disease severity: (1) *RFC1* disease without cerebellar involvement, which includes patients with isolated sensory neuropathy/neuropathy with bilateral vestibular areflexia, and (2) *RFC1* disease with cerebellar involvement, which includes patients with complex sensory and cerebellar ataxia or full CANVAS. Therefore, neuronal damage occurring within the cerebellum has been previously shown to be associated with higher elevation of NfL serum levels compared to neuropathies.^8^, ^9^, ^10^, ^11^, ^12^, ^13^, ^14^, ^15^, ^16^ Loss of independent ambulation was also considered as a marker of advanced disease.

Serum samples from age‐matched healthy controls (HC) were also collected from each participating center (London: 1 sample; Pavia: 13; Milan: 14; Paris: 4; Padova: 8; San Sebastian: 8). The assessment of HCs was based on medical history (through participants' interviews).

Blood sampling and storage were conducted following a standard operating procedure at each of the six different centers. In particular, blood was collected into serum separating tubes tubes and centrifuged at 20°C at 3500 rpm for 10 min. Serum was then aliquoted and stored at −20°C. Samples were anonymized and sent blinded for clinical details to the University College London (A.H., H.Z.) for analysis of NfL levels. Serum NfL concentration was measured using the Simoa NfL assay on an HD‐X analyzer (Quanterix, Billerica, MA, USA) in one round of experiments with one batch of reagents. Four quality control samples were run in duplicate; the mean intra‐assay coefficient of variation of duplicate determinations for concentration was 6.9%.

Statistical analysis was performed using SPSS, version 26.00 (IBM, Armonk, NY, USA) and GraphPad Prism 9.0.0 for Windows (GraphPad Software, San Diego, CA, USA).

Demographic and clinical data were described as mean (SD) or median (interquartile range) if normally or nonnormally distributed, respectively. Data normality was assessed using Q–Q plots and analytical tests (Kolmogorov–Smirnov test, Shapiro–Wilk test, and Anderson–Darling test), requiring consistent results from all tests to confirm normality.

Means between groups were compared using the *t* test for normally distributed data and the Mann‐Whitney *U* test for nonnormally distributed data. Correlations were assessed using Spearman's or Pearson's coefficients, as appropriate for data distribution. We conducted a multiple linear regression analysis to examine the relationship between serum NfL (dependent variable), cerebellar involvement, age at time of blood collection, and sex (independent variables).

The study protocol was approved by the local institutional review boards and ethics committees. Written informed consent was obtained from all patients and HCs. The study adhered to all applicable ethical regulations.

## Results

A total of 61 patients and 48 HCs were enrolled in the study. The demographics and clinical characteristics of participants are summarized in Table 1. There was no significant difference in the mean age at sample collection of the two groups (*P* = 0.13) or sex distribution (*P* = 0.823). Mean age at blood collection in patients with *RFC1* disease was 67.08 years (±10.70), with a mean age of onset of 55.16 years (±10.85) and a median disease duration of 11 years (6–15). Overall, 42 patients (69%) had signs and/or symptoms of cerebellar involvement. At the time of evaluation, 27 patients (44%) required walking aids. In 7 patients (11%) vestibular function was not assessed.

**TABLE 1:** Demographic details of patients and healthy control cohorts

Patient group	N	Age, y (min–max)	Sex, *F* (%)	NfL (pg/mL)
Controls	48	63.68 (37–79)	21 (44)	12.40 ± 4.64
*RFC1* disease	61	67.08 (38–86)	28 (46)	24.34 ± 9.24
*P*‐value		0.13	0.82	<0.0001
Without cerebellar involvement	19	64.11 (46–86)	6 (31)	21.84 ± 6.99
Isolated sensory neuropathy	11	66.91 (47–86)	4 (36)	24.48 ± 6.07
Sensory neuropathy with BVA	8	60.25 (46–77)	2 (25)	18.22 ± 6.84
With cerebellar involvement	42	68.42 (38–83)	22 (52)	27.88 ± 9.58
Complex sensory and cerebellar ataxia	9	65 (48–81)	6 (67)	26.69 ± 8.60
CANVAS	33	69.36 (38–83)	16 (48)	28.20 ± 9.94
*P*‐value		0.14	0.14	0.0081

Abbreviations: NfL, neurofilament light; BVA, bilateral vestibular areflexia; CANVAS, cerebellar ataxia, neuropathy, vestibular areflexia syndrome.

No significant difference in serum NfL levels was observed between stored samples and samples collected prospectively (23.18 vs. 26.62 pg/mL, *P* = 0.22).

**FIG. 1:**
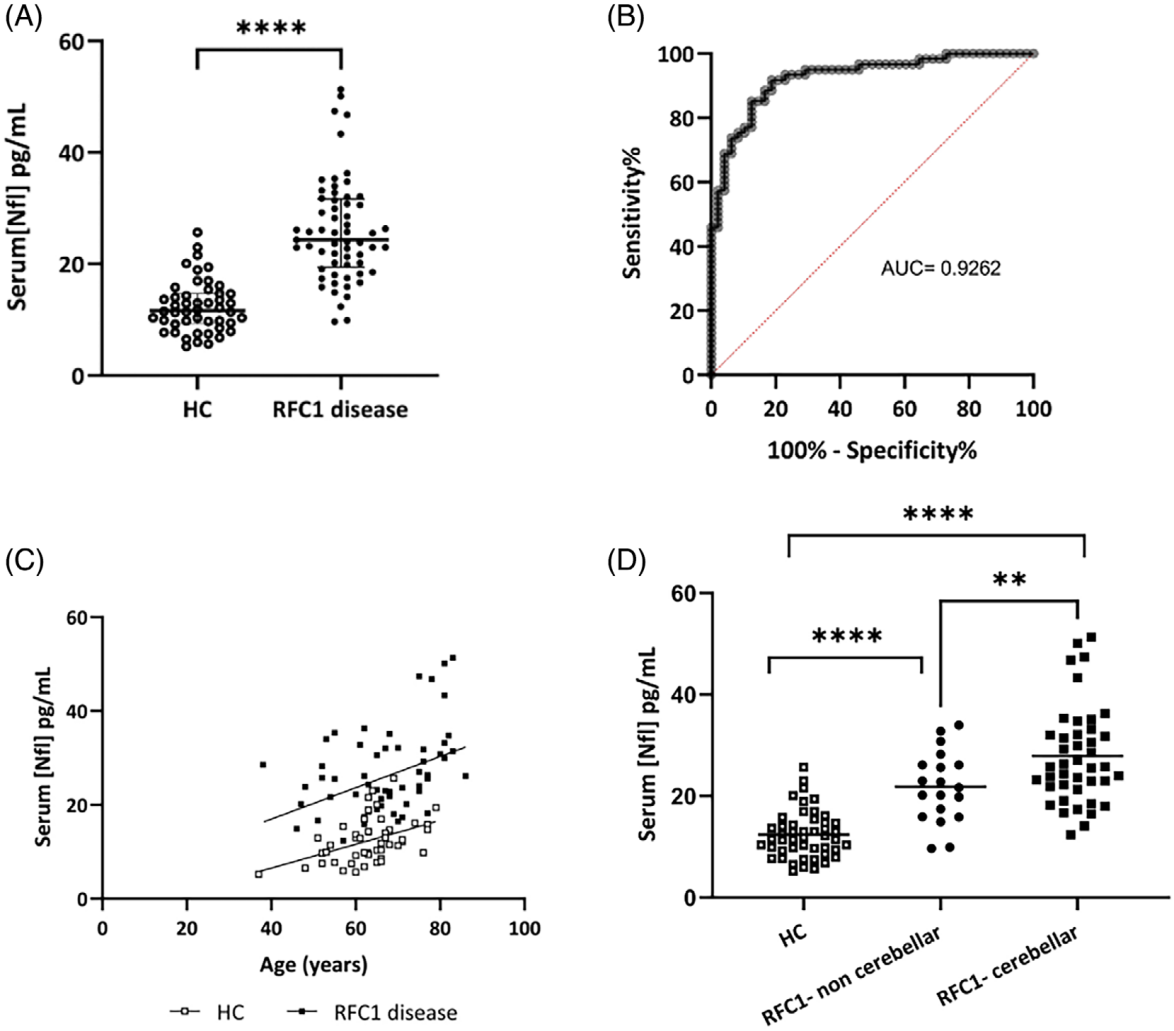
(**A**) Increased serum NfL concentration in patients with *RFC1* disease compared to healthy controls (HC). (**B**) Receiver operator curve of serum NfL concentration for detecting patients with *RFC1* CANVAS and disease spectrum. (**C**) Correlation between serum NfL concentration and age in both patients carrying *RFC1* expansions and HCs. (**D**) Increased serum NfL concentration in patients with cerebellar involvement compared to patients without cerebellar dysfunction. Both subgroups show higher concentrations compared to HCs. Line is at mean. Error bars: standard deviation. *P*‐value less than 0.05 is flagged with one star (*), *P*‐value less than 0.01 is flagged with two stars (**), and *P*‐value less than 0.001 is flagged with three stars (***). NfL: neurofilament light. CANVAS: cerebellar ataxia, neuropathy, vestibular areflexia syndrome. [Color figure can be viewed at wileyonlinelibrary.com]

Serum NfL concentration was significantly higher in patients with *RFC1* disease (24.34 pg/mL [IQR: 19.44–31.60]) compared to age‐matched HCs (12.40 pg/mL ± 4.64) (*P* < 0.0001; Fig. 1A).

Receiver operating characteristic analysis showed that serum NfL levels could discriminate patients from controls with great accuracy (AUC of 0.9262, 95% CI [confidence interval]: 0.88–0.97) (Fig. 1B). A concentration of 15.86 pg/mL can effectively identify individuals with *RFC1* disease with a sensitivity of 92% and a specificity of 81%.

Serum NfL concentration showed a moderate correlation with age in both HCs (*r* = 0.4353, *P* = 0.0020) and patients (*r* = 0.4092, *P* = 0.0011) (Fig. 1C).

The significant difference in serum NfL concentration compared to controls was maintained for individual comparisons of controls versus patients without cerebellar involvement (*P* < 0.0001), and of controls versus patients with cerebellar involvement (*P* < 0.0001).

Also, mean NfL concentrations were significantly higher in patients with cerebellar involvement compared to patients without cerebellar dysfunction (27.88 vs. 21.84 pg/mL, *P* = 0.0081) (Fig. 1D), and the association remained significant after controlling for age and sex in a multiple linear regression model (β = 0.260, 95% CI: 0.411002, *P* = 0.034).

Conversely, there was no significant difference in NfL levels between patients without signs of vestibular dysfunction (n = 13) and patients with vestibular impairment (n = 41) (25.28 vs. 26.25 pg/mL, *P* = 0.7061). Serum NfL levels did not appear to correlate with disease duration (*r* = 0.014, *P* = 0.917) and did not differ between patients with independent walking (n = 42) and patients using walking aid (n = 27) (24.12 vs. 28.36 pg/mL, *P* = 0.0820).

One patient had clinically manifest parkinsonism, with an NfL value (31.78 pg/mL) above the 75th percentile, and 3 patients presented with clinical signs of dysautonomia (mean NfL: 28.82 ± 6.26 pg/mL). Cognitive impairment was not reported in any patients.

## Discussion

This study is the first to investigate serum NfL levels as a biomarker in *RFC1* disease using ultrasensitive Simoa technology. We found significantly higher serum NfL levels in patients with biallelic *RFC1* expansions compared to HCs of the same age and sex. Elevated NfL levels were observed in various clinical phenotypes, including isolated sensory neuropathy, which is more common in early disease stages. NfL levels demonstrated excellent discriminatory power, supporting its potential as a reliable biomarker in various neurodegenerative ataxias,^12^, ^13^, ^17^, ^18^, ^19^ as well as in hereditary neuropathies.^8^, ^10^

NfL is an axonal cytoskeletal protein released after axonal damage. Abnormal NfL serum levels in our patients reflect the pathology and progression of *RFC1* disease. Postmortem examination revealed loss of sensory neurons in the dorsal root ganglia and Scarpa's ganglion, as well as marked loss of cerebellar Purkinje and granular cells.^14^, ^20^, ^21^, ^22^ A recent brain magnetic resonance imaging study also showed basal ganglia and brainstem volumetric reduction and involvement of the cerebral white matter in cases with advanced disease,^23^ suggesting a widespread cerebral neurodegeneration.

We have also demonstrated that patients with cerebellar damage have significantly higher levels of serum NfL than those without cerebellar involvement. This correlation persisted after correcting for age and gender. This may be explained by the high density of neurons in cerebellum, which would lead to a significantly increased release of NfL in the bloodstream when this structure is affected.

Conversely, serum NfL did not appear to correlate with disease duration and the need for walking aids. An explanation of this phenomenon is that NfL may increase rapidly in the initial stages of the disease and then reach a plateau above a certain degree of severity, as observed in other genetic ataxias.^12^, ^13^, ^18^ Other possible explanations entail the difficulty in accurately defining the onset of the disease, because neuropathy symptoms may remain unnoticed for a long time, or the chronologically variable involvement of the cerebellum in early or late disease stages relative to onset, due to factors yet to be explored, including the repeat size and additional genetic modifiers. Also, no significant difference in serum NfL levels was found based on vestibular involvement. This could be due to the limited quantitative relevance of Scarpa's ganglion, which contains around 20,000 neurons, compared to the cerebellum's 50 billion neurons.

This study has some limitations. First, the small sample size limits the statistical power of the study. Second, the relatively wide range in the time interval between serum collection and neurological examination (within 24 months at the most) may have introduced variability. However, previous studies in patients with spino‐cerebellar ataxias and Charcot–Marie–Tooth disease showed no significant difference in serum NfL concentration after 1 or 2 years,^8^, ^24^ suggesting stability of NfL levels in the short time in patients with slowly progressive neurodegenerative diseases. Finally, the cross‐sectional design of the study restricts causality assumptions and does not allow us to assess changes in serum NfL levels over time.

In conclusion, we have demonstrated that serum NfL holds promise as a reliable biomarker in *RFC1* disease, as NfL levels are elevated even in the early stages of the disease and exhibit a correlation with cerebellar involvement. Given the significant interindividual variability, NfL may prove to be a valuable tool for stratifying patients, and longitudinal studies are warranted to assess its ability to monitor disease progression.

## Author Roles

I.Q.: drafting and revision of the manuscript; major role in the acquisition, analysis, and interpretation of data. E.V.: revision and critique of the manuscript; major role in the acquisition, analysis, and interpretation of data. R.C.: revision and critique of the manuscript; major role in the acquisition, analysis, and interpretation of data. A.H.: revision and critique of the manuscript; acquisition, analysis, and interpretation of data. C.P.: revision and critique of the manuscript; acquisition, analysis, and interpretation of data. P.I.: revision and critique of the manuscript; acquisition, analysis, and interpretation of data. A.S.: revision and critique of the manuscript; acquisition, analysis, and interpretation of data. G.F.‐E.: revision and critique of the manuscript; acquisition, analysis, and interpretation of data. N.D.: revision and critique of the manuscript; acquisition, analysis, and interpretation of data. B.R.: revision and critique of the manuscript; acquisition, analysis, and interpretation of data. A.M.: revision and critique of the manuscript; acquisition, analysis, and interpretation of data. E.A.: revision and critique of the manuscript; acquisition, analysis, and interpretation of data. S.F.: revision and critique of the manuscript; acquisition, analysis, and interpretation of data. K.M.: revision and critique of the manuscript; acquisition, analysis, and interpretation of data. I.A.: revision and critique of the manuscript; acquisition, analysis, and interpretation of data. R.L.: revision and critique of the manuscript; acquisition, analysis, and interpretation of data. A.M.R.: revision and critique of the manuscript; acquisition, analysis, and interpretation of data. A.P.: revision and critique of the manuscript; acquisition, analysis, and interpretation of data. G.C.: revision and critique of the manuscript; acquisition, analysis, and interpretation of data. P.S.: revision and critique of the manuscript; acquisition and interpretation of data. E.S.: revision and critique of the manuscript; acquisition and interpretation of data. F.A.: revision and critique of the manuscript; acquisition and interpretation of data. E.M.V.: revision and critique of the manuscript; acquisition and interpretation of data. H.Z.: revision and critique of the manuscript; acquisition and interpretation of data. P.G.: revision and critique of the manuscript; acquisition and interpretation of data. T.S.: revision and critique of the manuscript; acquisition and interpretation of data. C.B.: revision and critique of the manuscript; acquisition and interpretation of data. A.L.M.: revision and critique of the manuscript; acquisition and interpretation of data. D.P.: revision and critique of the manuscript; acquisition and interpretation of data. M.M.R.: revision and critique of the manuscript; acquisition and interpretation of data. H.H.: revision and critique of the manuscript; acquisition and interpretation of data. C.T.: revision and critique of the manuscript; acquisition and interpretation of data. A.C.: study conceptualization, revision and critique of the manuscript, acquisition and interpretation of data. All authors have read and approved the final version of the manuscript.

## Full financial disclosures for the previous 12 months

The authors report no disclosures.

## Supporting information


**Data S1.** Supporting Information.

## Data Availability

Anonymized data from this study will be shared by request from any qualified investigator.

## References

[1] Cortese A , Simone R , Sullivan R , et al. Biallelic expansion of an intronic repeat in RFC1 is a common cause of late‐onset ataxia. Nat Genet 2019;51(4):649–658. 30926972 10.1038/s41588-019-0372-4 PMC6709527

[2] Rafehi H , Szmulewicz DJ , Bennett MF , Sobreira NLM , Pope K , Smith KR , et al. Bioinformatics‐based identification of expanded repeats: a non‐reference intronic pentamer expansion in RFC1 causes CANVAS. Am J Hum Genet 2019;105(1):151–165. 31230722 10.1016/j.ajhg.2019.05.016 PMC6612533

[3] Cortese A , Tozza S , Yau WY , et al. Cerebellar ataxia, neuropathy, vestibular areflexia syndrome due to RFC1 repeat expansion. Brain 2020;143(2):480–490. 32040566 10.1093/brain/awz418 PMC7009469

[4] Kulshreshtha D , Ganguly J , Jog M . Expanding the clinical Spectrum of RFC1 gene mutations. J Mov Disord 2022;15(2):167–170. 35306791 10.14802/jmd.21117 PMC9171309

[5] Record CJ , Alsukhni RA , Currò R , et al. Severe distinct dysautonomia in RFC1‐related disease associated with parkinsonism. J Peripher Nerv Syst 2022;27(4):311–315. 36177974 10.1111/jns.12515 PMC10092280

[6] Ylikotila P , Sipilä J , Alapirtti T , et al. Association of biallelic RFC1 expansion with early‐onset Parkinson's disease. Eur J Neurol 2023;30(5):1256–1261. 36705320 10.1111/ene.15717

[7] Ashton NJ , Janelidze S , Al Khleifat A , et al. A multicentre validation study of the diagnostic value of serum neurofilament light. Nat Commun 2021;12:3400. 34099648 10.1038/s41467-021-23620-z PMC8185001

[8] Sandelius Å , Zetterberg H , Blennow K , et al. Serum neurofilament light chain concentration in the inherited peripheral neuropathies. Neurology 2018;90(6):e518–e524. 29321234 10.1212/WNL.0000000000004932 PMC5818017

[9] Millere E , Rots D , Simrén J , et al. Plasma neurofilament light chain as a potential biomarker in Charcot‐Marie‐tooth disease. Eur J Neurol 2021;28(3):974–981. 33340200 10.1111/ene.14689

[10] Ticau S , Sridharan GV , Tsour S , et al. Neurofilament light chain as a biomarker of hereditary transthyretin‐mediated amyloidosis. Neurology 2021;96(3):e412–e422. 33087494 10.1212/WNL.0000000000011090 PMC7884985

[11] Maalmi H , Strom A , Petrera A , et al. Serum neurofilament light chain: a novel biomarker for early diabetic sensorimotor polyneuropathy. Diabetologia 2023;66(3):579–589. 36472640 10.1007/s00125-022-05846-8 PMC9892145

[12] Peng L , Wang S , Chen Z , et al. Blood neurofilament light chain in genetic ataxia: a meta‐analysis. Mov Disord 2022;37(1):171–181. 34519102 10.1002/mds.28783

[13] Wilke C , Mengel D , Schöls L , et al. Levels of neurofilament light at the Preataxic and ataxic stages of spinocerebellar ataxia type 1. Neurology 2022;98(20):e1985–e1996. 35264424 10.1212/WNL.0000000000200257 PMC9162044

[14] Traschütz A , Wilke C , Haack TB , et al. Sensory axonal neuropathy in RFC1‐disease: tip of the iceberg of broad subclinical multisystemic neurodegeneration. Brain 2022;145(3):e6–e9. 35230382 10.1093/brain/awac003

[15] Schoeberl F , Abicht A , Kuepper C , et al. Sensory neuropathy due to RFC1 in a patient with ALS: more than a coincidence? J Neurol 2022;269(5):2774–2777. 34821988 10.1007/s00415-021-10835-9 PMC9021066

[16] Chelban V , Nikram E , Perez‐Soriano A , et al. Neurofilament light levels predict clinical progression and death in multiple system atrophy. Brain 2022;145(12):4398–4408. 35903017 10.1093/brain/awac253 PMC9762941

[17] Yang L , Shao YR , Li XY , et al. Association of the Level of neurofilament light with disease severity in patients with spinocerebellar ataxia type 2. Neurology 2021;97(24):e2404–e2413. 34706976 10.1212/WNL.0000000000012945 PMC8673719

[18] Tezenas du Montcel S , Petit E , Olubajo T , et al. Baseline clinical and blood biomarker in patients with Preataxic and early‐stage disease spinocerebellar ataxia 1 and 3. Neurology 2023;100(17):e1836–e1848. 36797067 10.1212/WNL.0000000000207088 PMC10136009

[19] Donath H , Woelke S , Schubert R , et al. Neurofilament light chain is a biomarker of neurodegeneration in ataxia telangiectasia. Cerebellum 2022;21(1):39–47. 33893614 10.1007/s12311-021-01257-4 PMC8885493

[20] Montaut S , Diedhiou N , Fahrer P , et al. Biallelic RFC1‐expansion in a French multicentric sporadic ataxia cohort. J Neurol 2021;268(9):3337–3343. 33666721 10.1007/s00415-021-10499-5

[21] Huin V , Coarelli G , Guemy C , et al. Motor neuron pathology in CANVAS due to RFC1 expansions. Brain 2022;145(6):2121–2132. 34927205 10.1093/brain/awab449

[22] Reyes‐Leiva D , Aldecoa I , Gelpi E , Rojas‐García R . Motor neuron involvement expands the neuropathological phenotype of late‐onset ataxia in RFC1 mutation (CANVAS). Brain Pathol 2022;32(4):e13051. 35001451 10.1111/bpa.13051 PMC9245944

[23] Matos PCAAP , Rezende TJR , Schmitt GS , et al. Brain structural signature of RFC1‐related disorder. Mov Disord 2021;36(11):2634–2641. 34241918 10.1002/mds.28711

[24] Coarelli G , Darios F , Petit E , et al. Serum neurofilament light chain predicts cerebellar atrophy and clinical progression in spinocerebellar ataxia. Neurobiol Dis 2021;153:105311. 33636389 10.1016/j.nbd.2021.105311

